# Infection of endothelial cells by *Streptococcus agalactiae* reveals potential role of PI-2b pilus on endothelial barrier dysfunction

**DOI:** 10.1590/0074-02760250077

**Published:** 2025-11-17

**Authors:** Jessica Silva Santos de Oliveira, Bruna Alves da Silva Pimentel, Leonardo Nagao Ferreira, Maria Eduarda Negreiro e Silva, Gabriela da Silva Santos, Prescilla Emy Nagao

**Affiliations:** 1 Universidade do Estado do Rio de Janeiro, Instituto de Biologia Roberto Alcantara Gomes, Laboratório de Biologia Molecular e Fisiologia de Estreptococos, Rio de Janeiro, RJ, Brasil; 2 Fundação Técnico-Educacional Souza Marques, Escola de Medicina Souza Marques, Rio de Janeiro, RJ, Brasil

**Keywords:** Streptococcus agalactiae, endothelial cells, fibrinogen, VE-cadherin, cell permeability

## Abstract

**BACKGROUND:**

*Streptococcus agalactiae* is responsible for sepsis and meningitis, and the major cause of neonatal morbidity and mortality. However, how *S. agalactiae* disrupts endothelial barriers is poorly understood.

**OBJECTIVES:**

Analyse the influence of endothelial cell (HUVECs) growth under static and shear stress conditions during infection with *S. agalactiae*, and the role of pilus PI-2b during endothelial barrier disruption and increased endothelial permeability.

**METHODS:**

HUVECs under static and shear conditions were infected by *S. agalactiae* (GBS90356 and GBS90356*Δpilus2b*) strains in the presence and absence of fibrinogen. VE-cadherin was evaluated by immunofluorescence and RT-PCR assays, and the endothelial permeability by transwell assay.

**FINDS:**

Shear stress induced the alignment of HUVECs and increased the adherence of *S. agalactiae* strains (GBS90356 and GBS90356*Δpilus2b*), mainly in the presence of fibrinogen, in addition to greater peripheral localisation of VE-cadherin. Rupture points and damage to endothelial integrity was visualised after infection with the GBS90356WT strain, mainly in the presence of fibrinogen. RT-PCR analyses identified increase in VE-cadherin expression in HUVECs under shear stress and a decrease in VE-cadherin after infection, with increased levels of endothelial permeability.

**MAIN CONCLUSION:**

Data demonstrate for the first time the dysfunction of the adhesive barrier induced by the *S. agalactiae* ST-17 strain, mainly in HUVECs under shear stress, where PI-2b expression was essential to optimise the damage to endothelial integrity.

*Streptococcus agalactiae* is an important human pathogen with impacts on health and economy worldwide, being the main cause of maternal and neonatal infections, including chorioamnionitis, postpartum endometritis, pneumonia, sepsis and meningitis. *S. agalactiae* causes at least 400,000 maternal and neonatal infections, accounting for approximately 50,000 stillbirths and 50,000 to 100,000 infant deaths annually.^1^ In addition, *S. agalactiae* also promotes invasive infections in non-pregnant adults, especially in patients with comorbidities such as liver dysfunction, cancer, obesity, and diabetes.^2^ Sepsis caused by an aggressive inflammatory reaction of *S. agalactiae* can cause damage to multiple organ functions. Many bacteria produce virulence factors that can disrupt the endothelial barrier through a variety of mechanisms, including direct destruction of endothelial cells, alteration of the endothelial cytoskeleton, and disruption of cell-cell junctions during sepsis.^3^ Bacterial pilus plays a key role in immune activation and bacterial entry into the central nervous system.^4^ In *S. agalactiae*, pilus type 2b (PI-2b) mediates adhesion and invasion of brain endothelial cells and contributes to translocation across the blood-brain barrier.^5^ PI-2b also contributes to the pathogenesis of *S. agalactiae* infection by mediating invasion in several human epithelial cell lines (pulmonary A549, cervical HeLa, and colonic C2BBe1).^6^ ^,^ ^7^ In addition, the presence of PI-2b increased phagocytosis of *S. agalactiae* by murine and human macrophages.^8^ However, the pathway by which *S. agalactiae* crosses the endothelial barrier remains unclear.

Due to the luminal location, endothelial cells are specialised in detecting hemodynamic and mechanical changes, playing an essential role in maintaining blood fluidity, platelet aggregation and vascular tone. The important mechanical stimulus for endothelial cells is called wall shear stress, which is the frictional force exerted by blood flow applied tangentially to the endothelium. The most important mechanosensory complex is located at cell-cell junctions, which have been implicated as responsible for activating shear response pathways, including cell alignment and endothelial dysfunction.^9^ Although the static culture model is easier and cheaper compared to the shear stress model, the expression of surface molecules, morphology, cell signalling, and interactions with different pathogens can occur heterogeneously between the two models. Both the cells and molecules that pass through the blood vessel and the endothelial cells that line the vessel suffer the action of this force, which is caused by the viscosity of the blood tangentially to these cells. Endothelial dysfunction or injury leads to disruption of the microvascular barrier, resulting in increased extravascular fluid, tissue oedema, and death in septic patients.^10^

Breakdown of VE-cadherin-mediated endothelial barrier function leads to altered vascular permeability and remodelling of endothelial cells, which are associated with several disease processes. Furthermore, fibrinogen promotes leukocyte transendothelial migration in a VE-cadherin-dependent manner,^11^ and induces increased endothelial permeability to enable transendothelial cell migration.^12^ Fibrinogen fragments/degradation products are present at high levels in the plasma after traumatic injury or infections and can circulate via the bloodstream to distant vascular beds. Fibrinogen fragments resulted in disruption of endothelial barrier integrity, which was associated with a decrease in endothelial cells expression of VE-cadherin and increased cell permeability.^13^ In this work, we provide experimental evidence of the influence of shear stress during infection of HUVEC cells by *S. agalactiae*, as well as the role of PI-2b during endothelial barrier disruption and increased endothelial permeability.

## MATERIALS AND METHODS

*Bacterial strain origin and culture conditions* - *Streptococcus agalactiae* wild type (WT) GBS90356 (capsular type III, ST-17) and GBS90356∆*pilus2b* (pilus 2b deficient mutant of GBS90356 kindly provided by Dr Kelly Doran - University of Colorado Anschutz School of Medicine) strains were used in this study. GBS90356 strain was the first Brazilian ST-17 strain sequenced in Brazil by our group and was partially investigated for virulence properties.^14^ ^,^ ^15^ ^,^ ^16^ ^,^ ^17^ ^,^ ^18^ Both *S. agalactiae* strains were cultured on blood agar base plates containing 5% defibrinated sheep's blood (BAB; Sigma Aldrich, São Paulo, SP, Brasil) for 24 h at 37ºC. After, three colonies were grown in brain heart infusion broth (BHI; Sigma) at 37ºC until OD 540 reading of 0.4 [~10^8^ colony forming units (CFU) per mL].^19^

*Human umbilical vein endothelial cells (HUVECs)* - HUVECs were obtained by treating umbilical veins with 0.1% collagenase IV (Sigma). HUVECs were seeded in 25 cm^2^ flasks treated with porcine skin gelatin (Sigma) and cultured in medium 199 (M199 - Sigma) supplemented with 100 U mL^-1^ penicillin, 100 μg mL^-1^ streptomycin and 2.5 μg mL^-1^ amphotericin-B, 2 mM glutamine and 20% foetal bovine serum (FBS) at 37ºC and 5% CO_2_ until they reached confluence. Subsequently, confluent HUVECs monolayers (first or second passage) were treated with 0.025% trypsin/0.2% EDTA solution prepared in 0.01 M phosphate-buffered NaCl at pH 7.2 (PBS), rinsed in PBS, and used for experiments in culture plates (Corning, NY, USA).^19^

*Shear stress treatment* - HUVECs were submitted to steady laminar shear stress as previously described.^20^ Briefly, HUVECs were seeded at 8 x 10^5^ cells/well in regular six-well plates and allowed to reach confluence, typically in two-three days. HUVECs were exposed to 10 dyn/cm² of constant shear stress for 24 h on an orbital rotator (CO_2_ Resistant Shaker, Thermo Scientific™) using the equation τw = α√ρη(2πf)^3^, where τw is the shear stress, α is the radius of rotation (cm), ρ is the density of the liquid (g/L), η is ﬂuid viscosity (0.0075 dyn/cm² at 37ºC), and f is the rotation per second. The flow device was kept at 37ºC for 24 h in a 5% CO_2_ atmosphere. Each experimental condition was repeated three times. After shear stress, HUVECs monolayers were infected with *S. agalactiae* without orbital rotation as described below.

*Streptococcus agalactiae-HUVECs binding assays* - Confluent HUVECs cultured under static or shear stress conditions in tissue culture plates were infected with *S. agalactiae* (5 x 10^7^ CFU; MOI:100) in the presence or absence of fibrinogen (20 µg). Bacterial-cell contact was facilitated by centrifugation for 1 min at 200 g. Cells were then incubated at 37ºC in 5% CO_2_ for 1 h. Infected HUVECs were rinsed with PBS, and lysed with 0.5 mL of 25 mM Tris, 5 mM EDTA, 150 mM NaCl, and 1% Igepal (lysis buffer). Bacterial recovery was determined by plating samples on BAB medium. Data were expressed as the mean adherent CFU mL^-1^ of three experiments in triplicate.^19^

*Streptococcus agalactiae binding to immobilised fibrinogen* - Microtiter plates were coated overnight at 4ºC with 20 µg mL^-1^ of fibrinogen (Sigma). The plates were washed three times with 0.5% (v/v) Tween 20 in PBS (PBST). To block additional protein-binding sites, wells were treated for 1 h at room temperature with 200 μL of 1% BSA in PBS. *S. agalactiae* (~ 10^9^ CFU 200 µL^-1^) strains were added to triplicate wells of the microtiter plates and incubated for 1 h at room temperature. The plates were washed twice and stained with 0.5% (wt/vol) crystal violet. After, 50 mL of ethanol was added to each well to solubilise the dye. The OD at 550 nm was determined using a microplate reader. Each experiment was performed in triplicate, and the results were pooled and averaged.

*Immunofluorescence* - Endothelial cells infected with *S. agalactiae* strains (GBS90356WT or GBS90356∆*pilus2b*) in the presence or absence of fibrinogen (20 µg) were stained with VE-cadherin mouse mAb (1:50; Santa Cruz, São Paulo, Brasil), and secondary Alexa fluor 488 donkey anti-mouse or secondary Alexa fluor 647 goat anti-mouse (Santa Cruz) during 1 h at 37ºC. Coverslips were mounted on slides with fluorescent mounting medium containing 40,6-diamidino-2-phenylindole for staining of nuclei (ProLong; Invitrogen). Images were acquired with an inverted epifluorescence microscope Olympus IX71 TH4-100 model. VE-cadherin fluorescence intensity was quantified using the free image analysis software ImageJ (https://imagej.net/ij/index.html). For phalloidin staining, HUVECs were washed with PBS, fixed for 15 min with 4% paraformaldehyde at 4ºC, followed by incubation in 0.5% Triton X-100 solution for 5 min. The cytoskeleton was stained with phalloidin in the dark at room temperature for 30 min. The fluorescence images were observed under a fluorescence microscope. 

*Isolation of RNA and reverse transcriptase polymerase chain reaction (RT-PCR)* - HUVECs were collected by a cell-scraper after *S. agalactiae* infection, and total RNA was isolated using TRIzol reagent (Invitrogen, São Paulo, SP, Brazil) according to the manufacturer's protocol. The purity (A260/A280) and concentration of RNA were determined using a NanoDrop 2000 spectrophotometer (Thermo Fisher Scientific, São Paulo, SP, Brazil). Using the Maxima First Strand cDNA Synthesis Kit with dsDNase (Thermo Fisher Scientific), reverse transcription of cDNA was carried out in accordance with the manufacturer's instructions. Real-time reverse transcription PCR (RT-PCR) amplification was performed with SYBR Green Master Mix in a StepOne PCR amplifier (Thermo Fisher Scientific). Untreated HUVECs were used as the reference sample and β-actin was used as the endogenous control. The PCR primer sequences and annealing temperatures for PCR were: VE-cadherin (230 bp, Tm 55°C and 30 cycles) Forward 5'ACATCACAGTCAAGTATGGGC3' Reverse 5'GATGCAGAGTAAGATGGCTGC 3' and β-actin (289 bp, Tm 58°C and 25 cycles) Forward 5' TGGACTTCGAGCAAGAGATGG3' Reverse 5'ATCTCTTCTGCATCCTGTCG3' as described before.^21^ The amplified DNA products were separated on 2% agarose gel, stained with ethidium bromide, visualised and photographed. The assay was performed in triplicate, and each experiment was repeated at least three times.

*Permeability assay* - Sheared or static endothelial cells were plated at a density of 4 x 10^5^ cells mL^-1^ on the upper chamber of hanging inserts (Millicell, pore size of 0.4 µM; Millipore), and endothelial cell medium was added to the lower chamber. Following infection, FITC-dextran (250 µg mL^-1^; 40 kDa) was added to the endothelial cells in the upper chamber. Permeability was determined after 24 h by measuring the amount of FITC-dextran that permeated through the endothelial cells into the lower chamber with a fluorescent plate reader (1420 Victor V3; Perkin Elmer). The positive control corresponded to the translocation of FITC-dextran in the absence of HUVECs and bacteria. The negative control corresponded to monolayers of endothelial cells with intact adherent junctions, without bacteria. Data are expressed as percentages of 100% permeability.^22^

*Statistical analysis* - Data are presented as mean value and standard error of the mean (SEM) of at least three independent experiments. Differences between groups were tested using Student's t-test, in the case of bound groups; the paired t-test was applied. Differences were considered to be significant at p < 0.05.

*Ethics* - This project was approved by the Ethics Committee of the State Secretariat of Rio de Janeiro, receiving the number CAAE 51317415.8.3001.5259.

## RESULTS

**Fig. 1:**
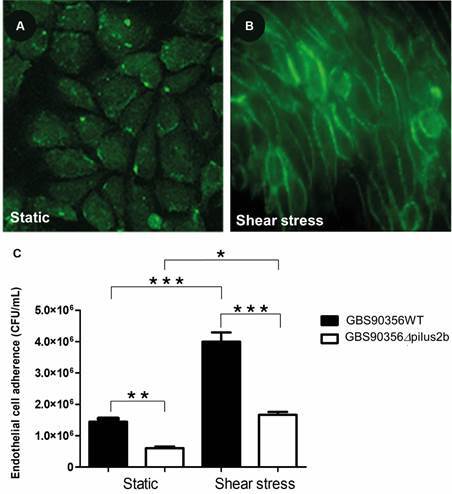
cell morphology of human umbilical vein endothelial cells (HUVECs). F-actin fluorescence analysis on HUVECs cultured in static (A) and *shear* stress (B) conditions. (C) Adherence of *Streptococcus agalactiae* (GBS9056WT and GBS90356*Δpilus2b*) on HUVECs under static and shear stress conditions. Results are presented as means ± standard error of the mean (SEM) from at least three independent experiments in triplicate wells. Asterisk indicates ^*^p < 0.02, ^**^p < 0.01, ^***^p < 0.001.

*Shear stress induces the alignment of HUVECs* - Phalloidin staining was performed to evaluate cell morphology of HUVECs in static and shear stress conditions. HUVECs in static model revealed a cobblestone morphology with no preferred orientation (Fig. 1A), whereas cells cultured under shear stress (10 dyn/cm^2^) showed a higher degree of cellular alignment along the direction of the flow (Fig. 1B).

*Increased adherence of S. agalactiae strains on HUVEC cultured under shear stress* - *Streptococcus agalactiae* strains were able to adhere to HUVECs monolayers cultured under static or shear stress conditions. The GBS90356WT strain showed higher adhesion capacity on HUVECs cultured under shear stress when compared to HUVECs in a static model after 1 h post-infection (p < 0.001; Fig. 1C). Similar results were observed for GBS90356*Δpilus2b* (p < 0.02; Fig. 1C). Furthermore, the GBS90356WT strain demonstrated higher adhesion in both models static (p < 0.01) and shear stress (p < 0.001) when compared to the PI-2b mutant strain (Fig. 1C).

**Fig. 2:**
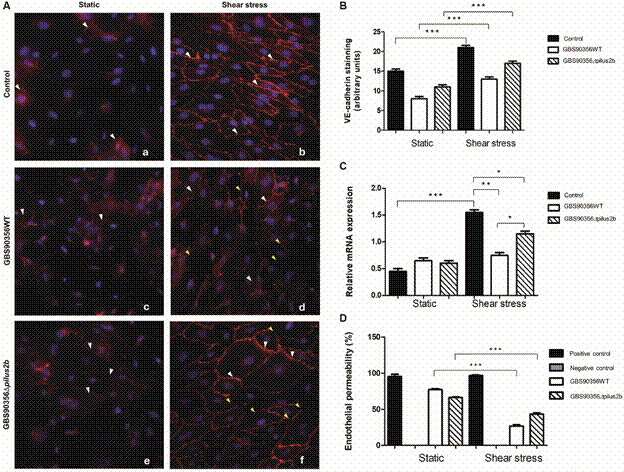
*Streptococcus agalactiae*-induced disturbances of VE-cadherin-mediated cell-cell interactions. (A) Immunofluorescent staining for VE-cadherin (red) and nuclei (blue) in human umbilical vein endothelial cells (HUVECs) under static (Aa, c, e) or shear stress conditions (Ab, d, f). HUVECs monolayer was stimulated with GBS9056WT (Ac, d) or GBS90356*Δpilus2b* (Ae, f) strains. (B) Quantification of VE-cadherin fluorescence intensity using ImageJ image analysis software. (C) mRNA expression in HUVECs were evaluated using real-time polymerase chain reaction (RT-PCR) after stimulation with *S. agalactiae* for 1 h. (D) HUVECs monolayer permeability was evaluated using the Millicell system. White arrowheads show intact VE-cadherin barrier, yellow arrowheads show VE-cadherin barrier disruption. Values are presented as means ± standard error of the mean (SEM) (n = 3) (^*^p < 0.02, ^**^p < 0.01, ^***^p < 0.001).

*Streptococcus agalactiae disrupts VE-cadherin junctions and increases endothelial permeability* - Immunofluorescence analysis showed a higher peripheral (membrane) localisation of VE-cadherin in HUVECs under shear stress compared to HUVECs cultured in static conditions (Fig. 2Aa-b). However, 1 h post-infection with bacterial strains, VE-cadherin labelling presented a diffuse and discontinuous pattern (Fig. 2Ac-f) with rupture points, showing a clear pattern of disruption of cell-cell junctions, and damage to the integrity of the endothelial monolayer under shear stress (Fig. 2Ad-f), mainly with the *S. agalactiae* GBS90356WT strain (Fig. 2Ad). Using imageJ software, we verified an increased VE-cadherin fluorescence in HUVEC exposure to shear stress (Fig. 2B; p < 0.001). The results were supported by RT-PCR analyses that identified significant increases in VE-cadherin expression in HUVECs subjected to shear stress compared to static HUVECs (Fig. 2B; p < 0.001). In contrast, both *S. agalactiae* strains induced a decrease of VE-cadherin expression in HUVECs under shear stress (Fig. 2B), mostly for GBS90356WT strain (Fig. 2B; p < 0.01). The results were consistent with endothelial barrier disruption, which the permeability levels were significantly increased in infected HUVECs compared to controls (Fig. 2D; p < 0.001).

**Fig. 3:**
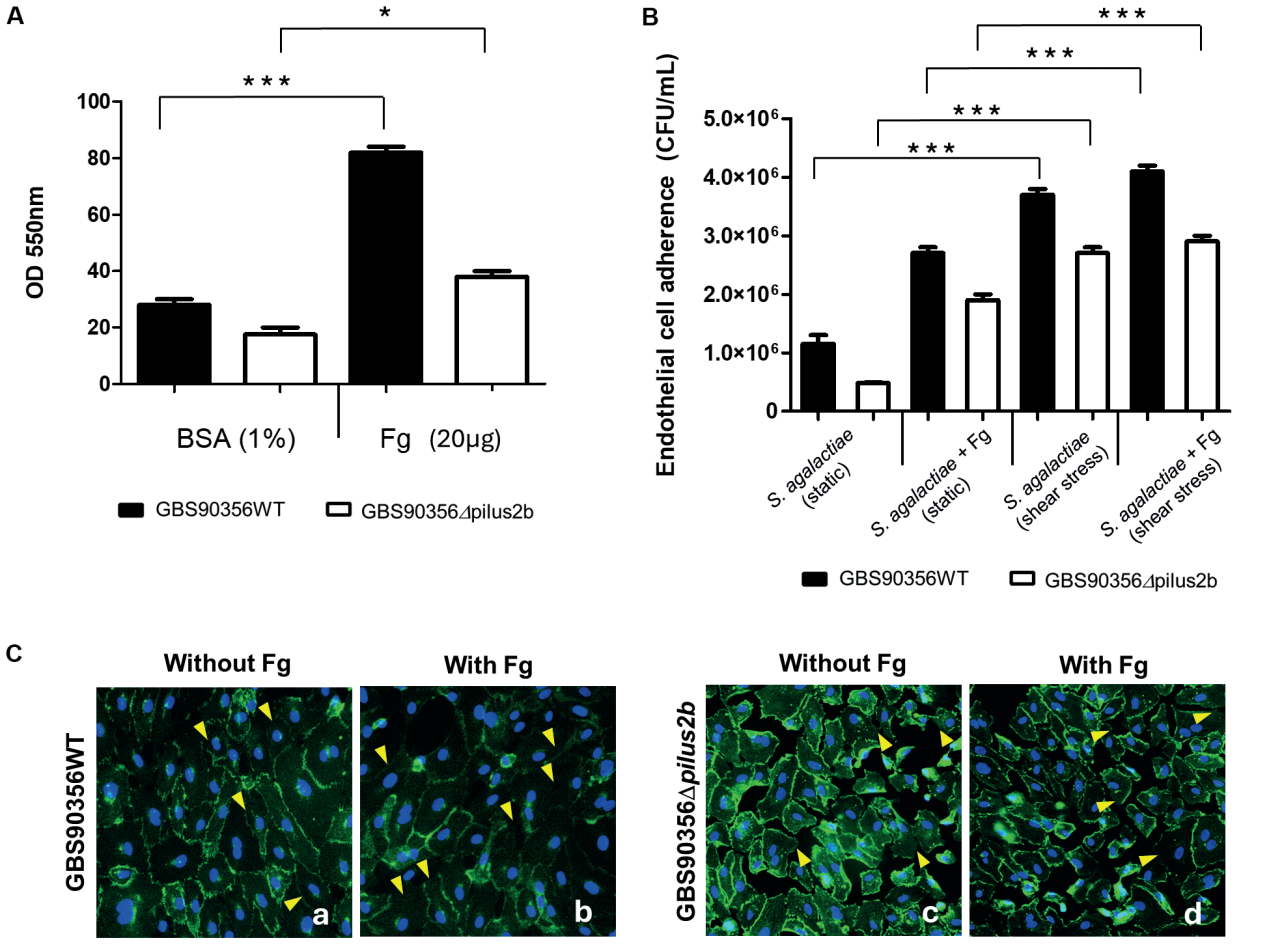
effect of fibrinogen on *Streptococcus agalactiae-*endothelial cell interaction. (A) *S. agalactiae* (GBS9056WT and GBS90356*Δpilus2b*) binding to immobilised fibrinogen (20 µg). (B) Fibrinogen enhances adherence of *S. agalactiae* in both human umbilical vein endothelial cells (HUVECs) grown in static or shear stress conditions. (C) Immunofluorescent staining for VE-cadherin (green) and nuclei (blue) in HUVECs under shear stress stimulated with GBS9056WT or GBS90356*Δpilus2b* in the presence of fibrinogen. White arrowheads show intact VE-cadherin barrier, yellow arrowheads show VE-cadherin barrier disruption. Data represent the mean ± standard error of the mean (SEM); ^*^p < 0.02, ^***^p < 0.001.

*Fibrinogen promotes binding of S. agalactiae strains to HUVECs* - Both *S. agalactiae* strains (GBS90356WT and GBS90356*Δpilus2b*) were able to adhere to immobilised fibrinogen, where the mutant showed lower potential when compared to the wild type strain (Fig. 3A; p < 0.001). To evaluate whether fibrinogen influences bacterial adhesion to HUVECs in response to endothelial cell growth conditions, bacterial-cell interaction was evaluated using HUVECs grown in static or under shear stress condition. In the presence of fibrinogen, the adhesive capacity of the bacterial strains was increased, especially in HUVECs cultured under shear stress. Furthermore, the GBS90356WT strain showed the highest adhesion in all conditions, especially in the presence of fibrinogen (Fig. 3B; p < 0.001). VE-cadherin staining showed several rupture points and numerous intercell spaces, demonstrating the participation of fibrinogen in the disruption of adherent's junctions, especially in HUVECs under shear stress infected by GBS90356WT strain (Fig. 3C; p < 0.001).

## DISCUSSION

The endothelium plays a key role during the development and progression of sepsis. Loss of the endothelial barrier can lead to increased leukocyte adhesion and transmigration, influx of large molecules and water across the endothelium, which can lead to multiple organ failure.^23^ Endothelial cells are exposed to shear stress, a fundamental physical characteristic of endothelial cell alignment, critical for vascular homeostasis.^24^ Our results confirmed that shear stress at physiological magnitudes is required for HUVECs to adopt an elongated and axially polarised phenotype in the flow direction, important for maintaining a functionally confluent cell monolayer. Based on this information, we performed the interaction of *S. agalactiae* strains with HUVECs under both static and shear stress growth conditions.

*Streptococcus agalactiae* ST-17 strains have been particularly well studied given their role as the major cause of neonatal disease, as well as the contribution of PI-2b to the virulence of ST-17 strains. PI-2b expression is regulated in GBS ST-17 strains by a 43-bp hairpin-like structure in the upstream region of PI-2b operon, conferring a selective advantage in the human host, either by reducing host immune responses or by increasing its dissemination potential. The PI-2b locus is found primarily in *S. agalactiae* type III ST-17, but may also be present in non-ST17 strains isolated from humans.^25^ Previous results suggested that high expression of the PI-2b locus in *S. agalactiae* strains could contribute to biofilm formation. The authors suggested that a complex regulation of PI-2b expression, indirectly mediated by CovR and other *S. agalactiae*-specific regulatory factors, would be involved in this process.^26^ Subsequently, the same authors showed that higher expression of the PI-2b pilus polymer resulted in increased phagocytosis by human monocyte-derived THP-1 macrophages.^7^ Moreover, the role of PI-2b in ST-17 pathogenesis by enhancing cell invasion, bacteraemia and spread into the central nervous system, suggested that this protein could be a therapeutic and prophylactic target against neonatal sepsis and meningitis caused by *S. agalactiae*.^5^

In the present work, there was a greater adhesive capacity of both *S. agalactiae* strains (GBS90356WT and GBS90356*Δpilus2b*) in HUVECs cultured in the shear stress than in the static condition. In addition, the GBS90356WT strain presented a higher viable bacteria count (CFU mL^-1^) when compared to the mutant strain for PI-2b, confirming the participation of this molecule during endothelial infection by this pathogen. *S. agalactiae* strains isolated from neonatal invasive infections in European countries showed high expression of PI-2b compared with PI-1.^27^ Expression of the PI-2b gene was two-fold higher in early-onset disease isolates compared to colonising isolates and mainly detected in capsular type III.^28^ The contribution of PI-2b to the virulence of ST-17 strains enhanced the efficiency of non-opsonic macrophage phagocytosis and conferred a survival advantage inside macrophages, promoting phagocyte resistance and systemic virulence.^29^ PI-2b also contributed to the initial attachment and invasion of *S. agalactiae* to cervical ME-180 and alveolar epithelial A549 cells.^30^ In agreement, our results demonstrated that the mutant *S. agalactiae* strain presented less adhesive capacity on HUVECs and less ability to disrupt junctions, when compared with GBS90356WT, showing the contribution of PI-2b to dissemination in the endothelial host cells.

Located in the basolateral regions of cells, the adherent junctions are responsible for maintaining cellular integrity. Among the cadherins, we can highlight the fundamental role of vascular endothelial cadherin (VE-cadherin), which directly regulates endothelial permeability and is also capable of binding to plasma proteins, increasing the capacity of the endothelium to respond to different stimuli from the extracellular environment.^31^ In our study, VE-cadherin upregulation was verified in HUVEC under shear stress and downregulation after infection by *S. agalactiae* strains, mainly for GBS90356WT. The decrease in VE-cadherin expression by the GBS90356*Δpilus2b* strain was lower when compared to the GBS90356WT strain, demonstrating the role of PI-2b during disease progression.

A previous study demonstrated that adherent's junction VE-cadherin controls tight junction organisation by promoting increased permeability. This mechanism occurs through upregulation of tight junction claudin-5, mediated by downregulation of VE-cadherin.^32^ The integrity of the blood-brain barrier was also increased after increased expression of claudin-5 and VE-cadherin in neurons.^33^ The link between adherents junction and tight junctions explains why VE-cadherin inhibition can cause a marked increase in permeability. Thus, we can suggest that VE-cadherin downregulation by *S. agalactiae* strains is an important pathogenic mechanism to increase endothelial permeability and promote bacterial dissemination during sepsis. In addition, both *S. agalactiae* strains (GBS90356WT and GBS90356*Δpilus2b*) caused VE-cadherin disruption, further increasing the invasive capacity of *S. agalactiae*, especially in the presence of PI-2b.

Some publications emphasise the importance of fibrinogen in the adhesion processes of *S. agalactiae* to host cells, including the surface proteins as FbsA, FbsB and FbsC,^34^ Srr1 and Srr2.^2^ In this work, the presence of *fbsA, fbsB, Srr1* and *Srr2* genes were detected in GBS90356 strain (data not shown). Consistently, *S. agalactiae* ST-17 clinical isolates exhibit a higher capacity to bind fibrinogen when compared with non-ST-17 strains.^35^ Srr2 has a higher affinity for fibrinogen than Srr1, suggesting that its expression may be critical for the hypervirulence of ST-17 isolates. Adhesion to fibrinogen is essential for early colonisation of host tissues and organs. Invading bacterial pathogens frequently bind to plasminogen/plasmin, and the resulting increase in cell surface proteolytic activity contributes to their subsequent dissemination within the infected host by facilitating the crossing of barriers such as the blood-brain barrier.^36^ Moreover, few studies reported the association between pili and fibrinogen. The contributions of the individual pilus subunits EmpA and EmpB during the adhesion of *Enterococcus faecium* to fibrinogen and type I collagen was demonstrated.^37^ Interestingly, significant reductions in adherence to fibrinogen and collagen type I were observed with deletion of *emp*A and *emp*B genes when compared to the wild type. Our results corroborate the previous data, where the absence of the pilus subunit (PI-2b) significantly reduced the binding of the *S. agalactiae* strains to fibrinogen, as well as the adhesive capacity on HUVECs, predominantly under shear stress conditions.

Although reductions in VE-cadherin expression and vascular barrier integrity following trauma and haemorrhage have been described, the mechanisms remain unknown.^38^ Various coagulation factors, including fibrinogen and/or fibrinogen degradation products, modulate the inflammatory response by affecting leukocyte migration and cytokine production.^39^ Vascular endothelial barrier disruption is a central mechanism in the development of multiple organ failure after severe trauma and haemorrhage. Fibrinolysis occurs during organ dysfunction, resulting in high circulating levels of fibrinogen degradation products, such as fragment X that induces endothelial cell hyperpermeability by reducing VE-cadherin expression. In addition to affecting VE-cadherin, fragment X also induced significant changes in genes that regulate endothelial cell-mediated coagulation, inflammation, angiogenesis, and vasoconstriction.^40^ Thus, fibrinogen and/or its fragments increased the severity of several inflammatory conditions. Presently, our results could suggest that *S. agalactiae* uses fibrinogen binding to induce changes in the expression of paracellular junctional proteins that regulate endothelial cell barrier function.

In conclusion, the fine-tuning PI-2b expression *in vivo* is essential to optimise *S. agalactiae* adherence to fibrinogen/fragment products and endothelial cells, favouring the breakdown of adherent junctions through VE-cadherin, and providing increased endothelial permeability for microorganism dissemination.
